# Developmental Changes in the Protein Expression Levels and Localization of the Vesicular Zinc Transporter (ZnT3) in the Mouse Central Auditory System

**DOI:** 10.3390/cells15171554

**Published:** 2026-08-27

**Authors:** Jesse Weisbord, Christopher L. Cunningham, Thanos Tzounopoulos, Brandon T. Bizup

**Affiliations:** Pittsburgh Hearing Research Center, Department of Otolaryngology, University of Pittsburgh, Pittsburgh, PA 15261, USA; jesseweisbord@gmail.com (J.W.); cunningc@pitt.edu (C.L.C.)

**Keywords:** zinc, ZnT3, auditory circuits, development

## Abstract

Vesicular (synaptic) zinc is a neuromodulator that fine-tunes synaptic transmission and sensory processing across many brain areas, including the brainstem, hippocampus, amygdala, and neocortex. Throughout the central auditory system, synaptic zinc plays a crucial role in modulating neurotransmission as well as baseline and adaptive sound processing. However, the developmental changes in the protein expression levels and localization of the vesicular zinc transporter (ZnT3), which loads synaptic zinc into presynaptic vesicles, remain unknown—due in part to the lack of robust ZnT3 antibodies. To address this question, we used the recently developed and validated ZnT3-HA transgenic mouse line, in which the ZnT3 protein contains a C-terminal HA epitope tag. We performed immunohistochemical staining and confocal microscopy in the central auditory system across development to localize and quantify changes in ZnT3 expression and explore potential colocalization of ZnT3 with the vesicular glutamate and GABA transporters VGLUT1 and VGAT, respectively. We found that ZnT3 expression increased significantly between P7 and P14 in both the dorsal cochlear nucleus (DCN) and the auditory cortex (AC), reaching a stable overall expression level and layer distribution by P21. Both in the DCN and AC, ZnT3 was mainly colocalized with VGLUT1. We did not find any significant levels of ZnT3 expression in either the IC or the auditory thalamus. Together, these results highlight major developmental changes in zinc signaling that may affect synaptic transmission and plasticity, as well as normal and pathological sound processing.

## 1. Introduction

Zinc is a biometal essential for life, and its deficiency is associated with numerous abnormalities such as stunted growth, weakened immunity, defective skin and bone formation and repair, and cognitive impairment [1]. Most of the zinc in the body is covalently bound to proteins, serving as a structural or catalytic molecule [2,3]. However, about 10% of zinc exists in an unbound state, where it serves as a crucial signaling molecule [4,5]. Specifically, synaptically released (synaptic) zinc is an important neuromodulator that is co-released with glutamate and acts mainly on glutamatergic, GABAergic and glycinergic receptors to fine-tune neurotransmission in the brainstem, hippocampus, neocortex and amygdala [6]. Namely, a significant subset of glutamatergic neurons contain high levels of synaptic zinc within their glutamatergic vesicles [7], which mainly modulates the activity of N-methyl-d-aspartate receptors (NMDARs), α-amino-3-hydroxy-5-methyl-4-isoxazolepropionic acid receptors (AMPARs), GABA receptors (GABARs) and glycine receptors [8,9,10,11,12,13,14,15]. Moreover, synaptic zinc levels are modulated bidirectionally by sensory experience [10,16,17,18], supporting roles in brain adaptation and plasticity. Indeed, through these actions synaptic zinc is a major neuromodulator that shapes baseline neurotransmission and synaptic plasticity, as well as neural processing and adaptation, especially in sensory systems [6,19].

Synaptic zinc is packaged into vesicles for synaptic release by the vesicular zinc transporter [20,21], ZnT3, which is found primarily in vesicles that express the vesicular glutamate transporter 1 (VGLUT1) [7]. Moreover, robust evidence over many years has demonstrated the distribution of synaptic zinc in the brain using histochemical stains (i.e., Timms staining) [22,23]. Although *Znt3* mRNA (*Slc30a3*) expression patterns are consistent with Timms staining [22], the spatiotemporal dynamics of the ZnT3 protein expression levels have been poorly understood, due to insufficient anti-ZnT3 antibody specificity, and by extension, the developmental trajectory of ZnT3 is mostly undescribed.

Here, to investigate the developmental changes in the expression of ZnT3 protein, we utilized a recently developed and validated transgenic mouse line that expresses ZnT3 with an epitope tag at the C-terminus of the endogenous *Slc30a3* gene (ZnT3-HA mouse) [18], thus allowing for reliable immunolabeling of ZnT3 protein using anti-HA antibodies [18]. Using the ZnT3-HA mouse, we investigated the expression pattern and levels of endogenously expressed ZnT3 in key central auditory structures including the dorsal cochlear nucleus (DCN), inferior colliculus (IC), auditory thalamus, and auditory cortex (AC) at several stages of postnatal development and in adulthood. We chose the central auditory system, because there have been extensive studies on the crucial role of synaptic zinc signaling in the auditory system, where synaptic zinc fine-tunes neurotransmission and sound processing [10,19,24,25,26,27,28]. We found that ZnT3 protein expression increases throughout development in a layer- and subregion-specific pattern. Moreover, co-labeling with antibodies against VGLUT1 and VGAT allowed us to investigate how ZnT3 is co-expressed in the auditory system with these presynaptic markers. Our results highlight developmentally regulated, auditory region-specific expression of ZnT3 in glutamatergic and GABAergic neurons in the central auditory system. These data provide insight into the dynamics of synaptic zinc signaling throughout auditory development and demonstrate the usefulness of the ZnT3-HA mouse in studying zinc neurobiology.

## 2. Materials and Methods

### 2.1. Animals

All mouse handling was approved by the Institutional Animal Care and Use Committee (IACUC) at the University of Pittsburgh. The approved IACUC protocol numbers that were used in this study were 23114004 and 23063309. Mice were maintained on a 12 h light cycle from 7 a.m. to 7 p.m. and were all a part of in-house colonies. Brains of mice were collected at five different ages: postnatal days (P) 7, P14, P21, P28, and P56/adult. ZnT3-HA mice were used for ZnT3 protein localization because commercially available anti-ZnT3 antibodies were not effective in our hands. The ZnT3-HA mouse contains a hemagglutinin (HA) tag at the carboxy terminus of the ZnT3 protein (Supplemental Figure S1) which allows us to use commercially available and reliable anti-HA antibodies to immunolabel the ZnT3-HA protein. This mouse line was created and validated as described, including the full genetic knock-in strategy, in a previous publication [18]. Wildtype littermate mice from the ZnT3-HA colony were used for the negative control experiments. These mice possess two copies of the wildtype *Znt3* gene and no copies of the *Slc30a3-HA* gene.

### 2.2. Tissue Collection and Cryosectioning

Mice were anesthetized and euthanized with 5% isoflurane prior to tissue extraction. Brains of P7, P14, and P21 mice were removed and fixed via immersion in 4% PFA for 48 h at 4 °C. P28 and P56 mice were fixed by transcardial perfusion using 1X PBS followed by 4% paraformaldehyde (PFA), dissection and post-fixed by immersion. To cryoprotect the tissue, brains were first placed into a 15% sucrose solution at 4 °C for 24 h, then into a 30% sucrose solution at 4 °C for 24 h, and finally into a 1X PBS solution containing 0.01% sodium azide at 4 °C. Brains were embedded in optimum cutting temperature (OCT) compound (Scigen Tissue-Plus, Fisher Scientific, Waltham, MA, USA, Cat.# 23-730-571) on a cryostat chuck and allowed to equilibrate to the temperature inside the cryostat for 20 min. Brains were then cryosectioned (CryoStar NX50 cryostat, Thermo Fisher Scientific, Waltham, MA, USA) into 35 µm thick slices, prepared at −20 °C for P14 and older mice, and at −20 °C for P7 mice. Slices were stored in a tissue well plate containing a 1X PBS with 0.01% sodium azide solution at 4 °C until staining. Tissue from each of the 5 age groups as well as 2 additional sets of wildtype tissue were sliced, stained, imaged, and analyzed together. For all analyses, 3 mice were used per age group. Within each mouse, 3 separate sections for each brain region (rostral, middle, and caudal) were selected for imaging and analysis, and the results were averaged within each mouse to generate a single data point. Per-mouse averages were used for all statistical tests.

### 2.3. Fluorescent Immunohistochemistry

The tissue was immunostained as free-floating slices in well plates. Slices were permeabilized by washing three times for 10 min in 1X PBS with 0.5% Triton-X 100 (0.5% PBST) and then blocked in 0.5% PBST with 5% normal goat serum (blocking solution) for 1 h at room temperature (RT). Next, tissue was incubated for 72 h at 4 °C in blocking solution containing primary antibodies (Rabbit IgG monoclonal anti-HA tag, 1:1000, Cell Signaling Technologies, Danvers, MA, USA, Cat.# 37245, RRID: AB_1549585; Guinea pig polyclonal anti-VGLUT1, 1:1000, Synaptic Systems, Göttingen, Germany, Cat.# 135304, RRID: AB_887878; Guinea pig polyclonal anti-VGAT, 1:1000, Synaptic Systems, Göttingen, Germany, Cat.# 131004, RRID: AB_887873; or Mouse IgG1 anti-calbindin, 1:2000, Swant, Kehrsatz, Switzerland, Cat.# 300, RRID: AB_10000347). Tissue was then washed three times for 10 min in 0.5% PBST at RT before being incubated for 24 h at 4 °C in the blocking solution containing the secondary antibodies (Goat anti-Rabbit IgG Alexa Fluor 555, 1:1000, Invitrogen, Waltham, MA, USA, Cat.# A21429, RRID: AB_2535850; Goat anti-Guinea pig IgG Alexa Fluor 488, 1:1000, Invitrogen, Waltham, MA, USA, Cat.# A11073, RRID: AB_2534117; and Goat anti-Mouse IgG Alexa Fluor 647, 1:1000, Invitrogen, Waltham, MA, USA, Cat.# A32728, RRID: AB_2633277) and DAPI (1:5000, Thermo Scientific, Waltham, MA, USA, Cat.# 62248). The next day the tissue was washed three times for 10 min in 1X PBS and mounted on slides with 50 µL of Vectashield HardSet Antifade Mounting Medium (Vector Laboratories, Newark, CA, USA, Cat.#H-1400-10).

### 2.4. Confocal Imaging and Analysis

Fluorescent images were captured using the Stellaris 5 Leica Confocal Microscope running Leica Application Suite (LASX, Leica Microsystems, Wetzlar, Germany, software version 4.5) in counting mode. Confocal settings were optimized automatically using the LASX (Leica Microsystems, Wetzlar, Germany, software version 4.5) software settings to maximize signal/noise ratio and minimize bleed-through (pinhole = 1.0 airy unit; z-step size for 63X images: 0.3 μm; 2X line averaging). Laser power settings were set using negative control tissue and maintained for each set of tissue. Analysis of 20x images was done using the LASX program. The negative control image was used to set the threshold for each channel, which was then applied to all images being analyzed. Analysis of the 63x images was done with ImageJ. The composite image was then split into the two individual channels—ZnT3-HA and VGLUT/VGAT. Next, each channel was auto-thresholded using the Li thresholding method to remove background and autofluorescence, and a mask of this threshold was created to isolate positive pixels. The “AND” operation was then used on the two masks, which created a third image that consists only of pixels that were positive in both channels, which we considered to be colocalized pixels. Colocalized images were analyzed using the “measure” function and the “% area” value, which reflects the percentage of pixels within the image that were positive for both fluorophores. Percent (%) colocalization was calculated to determine the percent of VGLUT1 or VGAT immunolabeling that colocalizes with ZnT3-HA immunolabeling. The % Colocalization was calculated by taking the % area value of the colocalized mask image, divided by the % area of either VGLUT1 or VGAT, multiplied by 100.

$$\%\;\mathrm{Colocalization}=\frac{\%\;area\;of\;colocalized\;image}{\%\;area\;of\;VGLUT1\;or\;VTAT}*100$$

To determine subregions of the medial geniculate body (MGB), we used the anti-calbindin antibody, which preferentially labels the medial and dorsal but not the ventral MGB [26]. The molecular layer and deep layers of the DCN were determined based on VGLUT1 and ZnT3-HA staining, as well as the density of DAPI-stained nuclei. Auditory cortex boundaries were estimated based on the location of the rhinal fissure and the hippocampus.

A limitation of our analysis is that brains from younger mice (P7–P21) were fixed by immersion, while brains from older mice (P28–P56) were fixed by transcardial perfusion. As such, we cannot exclude that this methodological difference might affect morphology, antigen preservation, and quantitative fluorescence intensity.

### 2.5. Statistical Analysis

For statistical analysis between multiple groups with a single factor (age), a one-way ANOVA was used. For statistical analysis between multiple groups with two factors (age and region), a two-way ANOVA was used. Multiple comparisons were done using the Bonferroni correction. Details of the results of the statistical tests can be found in the statistics table.

## 3. Results

**Figure 1:**
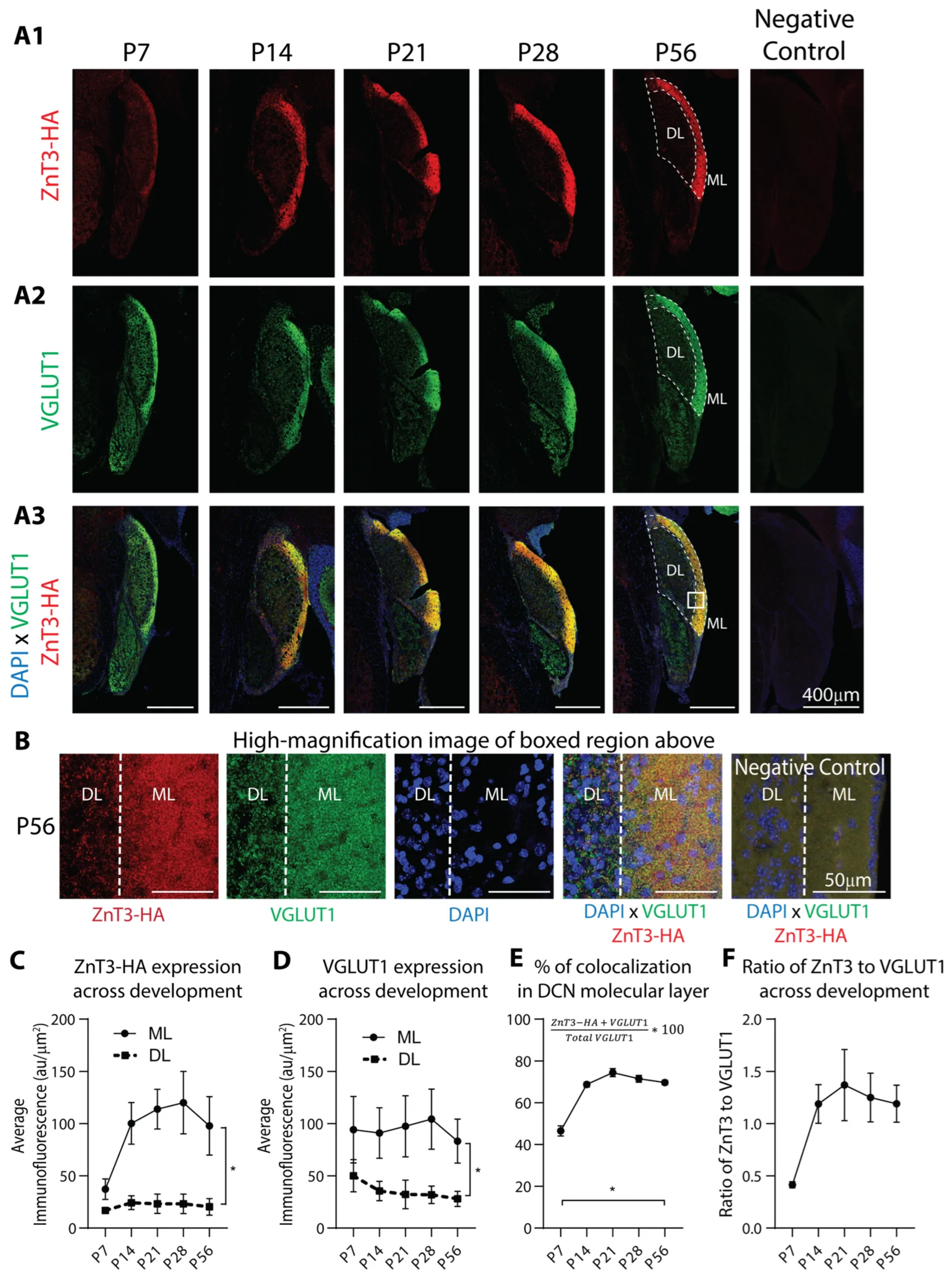
**Expression of ZnT3 and its colocalization with VGLUT1 in the DCN.** (**A1**) Representative low-magnification (20×) images of ZnT3-HA (red) staining in the DCN throughout development from P7 to P56. Negative control image from P56 mouse on far right. (**A2**) Representative low-magnification (20×) images of VGLUT1 (green) staining in the DCN throughout development from P7 to P56. Negative control image from P56 mouse on far right. (**A3**) Composite representative low-magnification (20×) images of ZnT3-HA, VGLUT1, and DAPI (blue) staining in the DCN throughout development from P7 to P56. Negative control image from P56 mouse on far right. Scale bar = 400 µm. (**B**) Representative high-magnification (63×) images of ZnT3-HA stain, VGLUT1 stain, DAPI stain, and the composite image (from left to right) in the DCN of a P56 mouse. Negative control image from p56 mouse on far right. Scale bar = 50 µm. (**C**) In reference to (**A1**). Quantification of average immunofluorescence of ZnT3-HA expression in the DL and ML of the DCN throughout development from P7 to P56. N = 3 mice per age group. Result of 2-way ANOVA: effect of age—*p* = 0.2649; effect of region—*p* < 0.0001 *; effect of age × region—*p* = 0.0658; post hoc effects of P7 v P21—*p* = 0.0305 and P7 v P28—*p* = 0.0174. (**D**) In reference to (**A2**). Quantification of average immunofluorescence of VGLUT1 expression in the DL and ML of the DCN throughout development from P7 to P56. N = 3 mice per age group. Result of 2-way ANOVA: effect of age—*p* = 0.9781; effect of region—*p* < 0.0001 *; effect of age X region—0.8180. (**E**) In reference to (**B**). Quantification for percentage of colocalization between ZnT3-HA and VGLUT1 expression in the ML of the DCN throughout development from P7 to P56. N = 3 mice per age group. Result of 1-way ANOVA: effect of age—*p* < 0.0001 *. (**F**) Ratio of ZnT3 to VGLUT1 expression in the ML of the DCN throughout development. For statistical details, see Table 1.

To investigate the spatial and developmental expression profile of the vesicular transporter ZnT3 in the central auditory nervous system, we employed a ZnT3-HA knock-in mouse that expresses a human influenza hemagglutinin (HA) epitope tag at the C-terminus of the endogenous ZnT3 (*Slc30a3*) gene. To visualize and measure ZnT3-HA (ZnT3) protein expression, we immunolabeled cryosections with anti-HA antibodies and counterstained nuclei with DAPI. We first investigated the DCN, where previous studies have uncovered robust synaptic zinc signaling [10,23,29]. We observed low levels of ZnT3 expression in the DCN molecular layer (ML) at P7, which increased at P14 and peaked at P28 (Figure 1A,C; Table 1). We did not observe any significant ZnT3 expression in the deep layer (DL; Figure 1A1,C). This expression pattern is consistent with previous histochemical studies assessing free zinc levels and electrophysiological studies assessing the effect of vesicular (synaptic) zinc on NMDARs and AMPARs in the DCN [8,10,11,12,23,29].

To assess colocalization with glutamatergic synapses, we co-stained sections with antibodies for ZnT3 and VGLUT1. With high-magnification confocal microscopic analysis, we observed considerable colocalization of ZnT3 with VGLUT1 in the DCN ML (Figure 1A3,B,D,E). Beginning at P7, we observed that 46.5% of ZnT3 puncta were also positive for VGLUT1, and this percentage increased to 68.7% by P14, after which the level of colocalization remained stable up to P56 (Figure 1E; Table 1). Lastly, we analyzed the ratio of ZnT3 to VGLUT1 expression over time in the DCN ML, and found that the ratio of ZnT3 to VGLUT1 increased from P7 to P14 (Figure 1F, Table 1). Given that ZnT3 expression increased during this time (Figure 1C), while VGLUT1 expression remained relatively stable (Figure 1D) and the colocalization increased (Figure 1E), these findings suggest an increase in the proportion of glutamatergic vesicles containing ZnT3.

**Figure 2:**
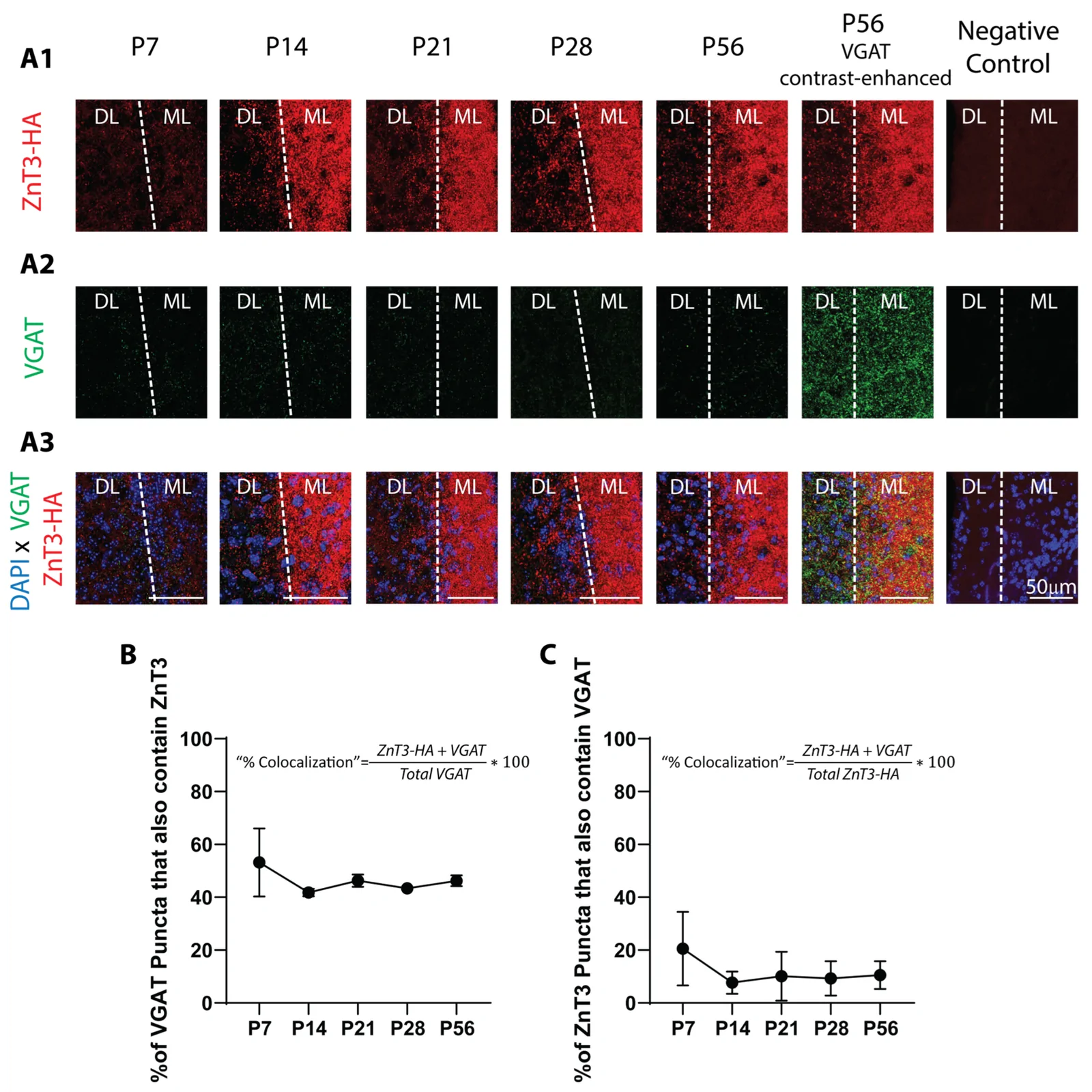
**Colocalization of ZnT3 with VGAT in the DCN.** (**A1**) Representative high-magnification (63×) images of ZnT3-HA (red) staining in the DCN throughout development from P7 to P56. Negative control image from P56 mouse on far right. (**A2**) Representative high-magnification (63×) images of VGAT (green) staining in the DCN throughout development from P7 to P56. P56 VGAT signal was contrast-enhanced for visualization only to highlight the extent of colocalization between VGAT and ZnT3-HA. Negative control image from P56 mouse on far right. (**A3**) Composite representative high-magnification (63×) images of ZnT3-HA, VGAT, and DAPI (blue) staining in the DCN throughout development from P7 to P56. P56 VGAT signal was also increased to help visualize the extent of colocalization between VGAT and ZnT3-HA. Negative control image from P56 mouse on far right. Scale bar = 50 µm. (**B**) In reference to (**A3**). Quantification for percentage of colocalization between ZnT3-HA and VGAT expression out of the total VGAT expression in the ML of the DCN throughout development from P7 to P56. N = 3 mice per age group. (**C**) In reference to (**A3**). Quantification for percentage of colocalization between ZnT3-HA and VGAT expression out of the total ZnT3-HA expression in the ML of the DCN throughout development from P7 to P56. N = 3 mice per age group. For statistical details, see Table 1.

We assessed ZnT3 colocalization with GABAergic synapses by co-staining section antibodies for ZnT3 and VGAT. We observed much lower expression levels of VGAT relative to VGLUT1. When we assessed colocalization of ZnT3-HA with VGAT, we found close to 50% colocalization which did not vary significantly across development (Figure 2A,B; Table 1). However, the colocalized ZnT3 and VGAT signal constitutes a low percentage of the overall ZnT3 signal, as only around 10% of the overall ZnT3 signal is colocalized with VGAT (Figure 2A,C; Table 1, Section 4). Taken together, these results demonstrate that ZnT3 is associated with the majority of DCN synapses at least as early as P7 and suggest that ZnT3 is primarily co-expressed in vesicles with VGLUT1, consistent with previous studies [7].

**Figure 3:**
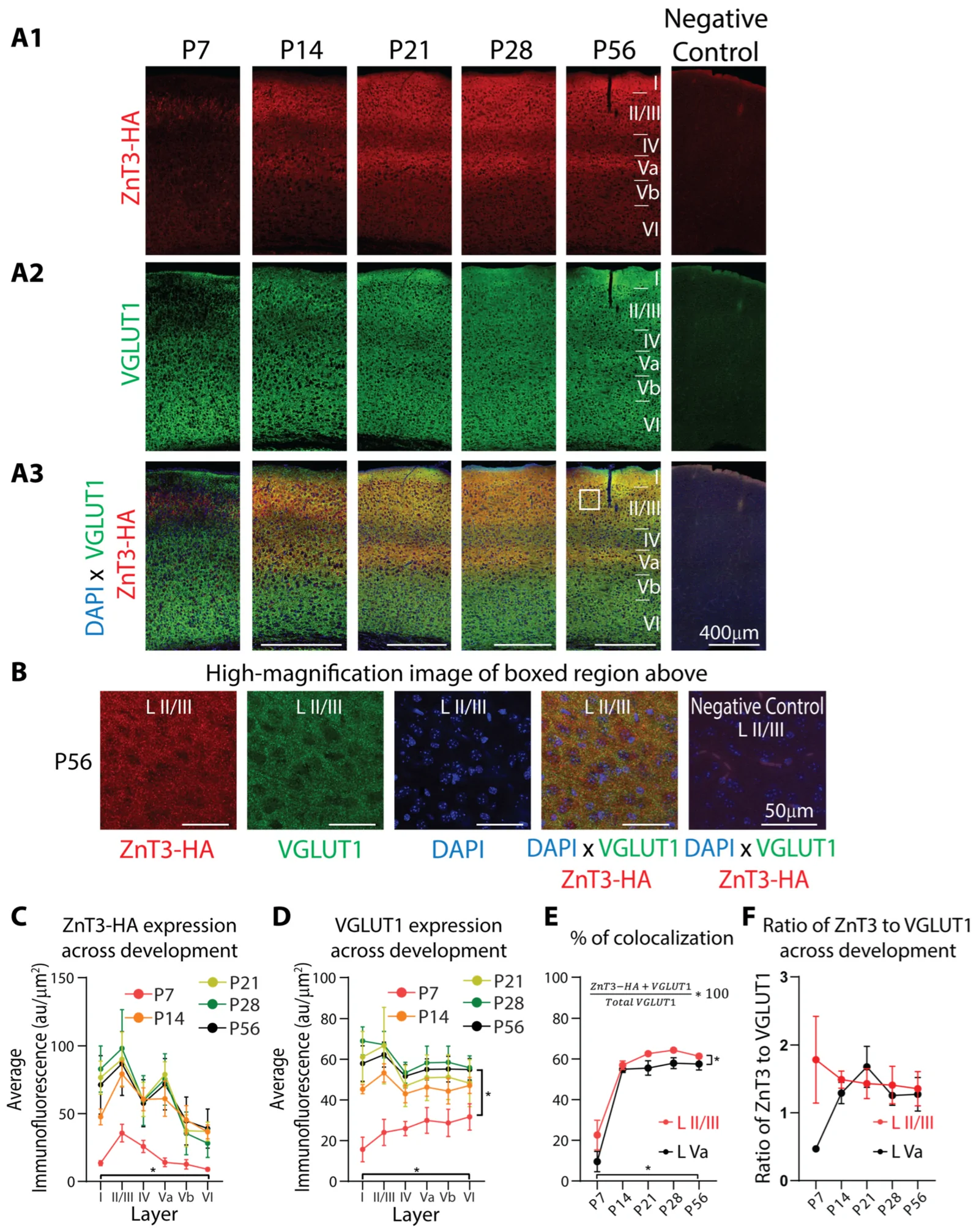
**Expression of ZnT3 and its colocalization with VGLUT1 in the AC.** (**A1**) Representative low-magnification (20×) images of ZnT3-HA (red) staining in the AC throughout development from P7 to P56. Negative control image from P56 mouse on far right. (**A2**) Representative low-magnification (20×) images of VGLUT1 (green) staining in the AC throughout development from P7 to P56. Negative control image from P56 mouse on far right. (**A3**) Composite representative low-magnification (20×) images of ZnT3-HA, VGLUT1, and DAPI (blue) staining in the AC throughout development from P7 to P56. Negative control image from P56 mouse on far right. Scale bar = 400 µm. (**B**) Representative high-magnification (63×) images of ZnT3-HA stain, VGLUT1 stain, DAPI stain, and the composite image (from left to right) in AC Layer II/III of a P56 mouse. Negative control image from p56 mouse on far right. Scale bar = 50 µm. (**C**) In reference to (**A1**). Quantification of average immunofluorescence of ZnT3-HA expression in the AC layers throughout development from P7 to P56. N = 3 mice per age group. Result of 2-way ANOVA: effect of age—*p* = 0.1430; effect of region—*p* < 0.0001 *; effect of age X region—0.0019 *. (**D**) In reference to (**A2**). Quantification of average immunofluorescence of VGLUT1 expression in AC layers throughout development from P7 to P56. N = 3 mice per age group. Result of 2-way ANOVA: effect of age—*p* = 0.0454 *; effect of region—*p* = 0.0144 *; effect of age X region—0.0211 *. (**E**) In reference to (**B**). Quantification for percentage of colocalization between ZnT3-HA and VGLUT1 expression in AC L II/III and V throughout development from P7 to P56. N = 3 mice per age group. Result of 2-way ANOVA: effect of age—*p* < 0.0001 *; effect of region—*p* = 0.0001 *; effect of age X region—0.0711. (**F**) Ratio of ZnT3 to VGLUT1 expression over time in AC Layer II/III (red) and Layer Va (black). For statistical details, see Table 1.

We next assessed the developmental expression of ZnT3 in the AC, where synaptic zinc signaling is associated with robust modulation of neurotransmission and sound processing [9,13,25,26,27,28,30,31]. At P7, we observed faint ZnT3 expression in superficial layers 2/3 (L2/3) (Figure 3A,C), when AC laminar organization and connectivity in mice are not yet fully developed [32,33,34]. At P14, we observed higher levels of ZnT3-HA expression, and sections now resembled the typical AC laminar organization (Figure 3A1,C; Table 1). Namely, at P14 a second band of ZnT3 expression emerged in L5, and the ZnT3 immunolabeling located in L2/3 expanded (Figure 3A,C). By P21, the laminar distribution of ZnT3 closely resembled the known distribution of vesicular zinc, with ZnT3 primarily expressed in L2/3 and 5a (Figure 3A,C, Table 1) [6,22]. The delayed increase in L5 ZnT3 expression at P21 relative to other layers (e.g., L2/3), suggests that synaptic zinc signaling maturation varies based on cortical layer.

**Figure 4:**
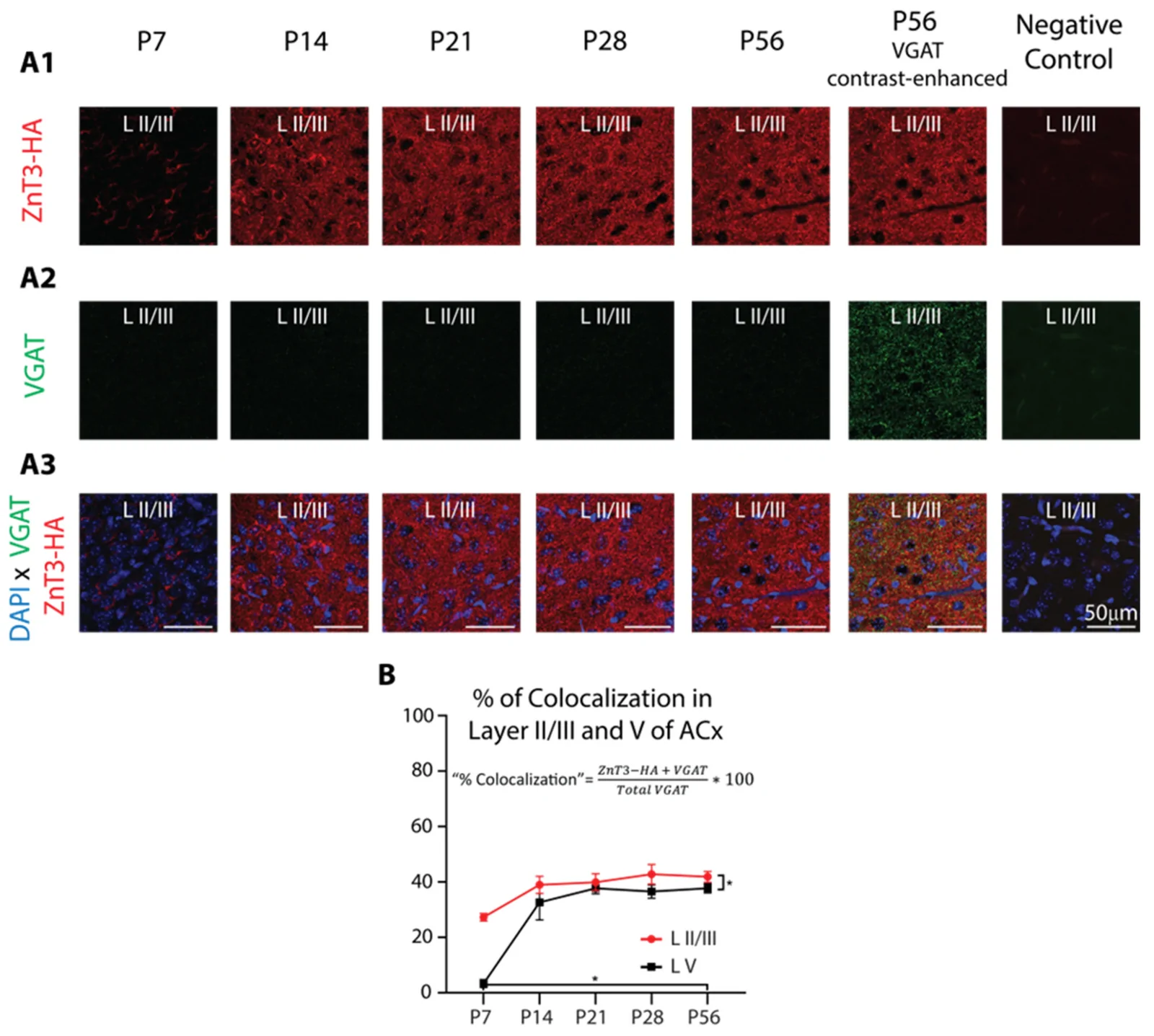
**Colocalization of ZnT3 with VGAT in the AC.** (**A1**) Representative high-magnification (63×) images of ZnT3-HA (red) staining in AC LII/III throughout development from P7 to P56. Negative control image from P56 mouse on far right. (**A2**) Representative high-magnification (63×) images of VGAT (green) staining in in AC LII/III throughout development from P7 to P56. P56 VGAT signal was contrast-enhanced for visualization only to highlight the extent of colocalization between VGAT and ZnT3-HA. Negative control image from P56 mouse on far right. (**A3**) Composite representative high-magnification (63×) images of ZnT3-HA, VGAT, and DAPI (blue) staining in AC LII/III throughout development from P7 to P56. P56 VGAT signal was also increased to help visualize the extent of colocalization between VGAT and ZnT3-HA. Negative control image from P56 mouse on far right. Scale bar = 50 µm. (**B**) In reference to (**A3**). Quantification for percentage of colocalization between ZnT3-HA and VGAT expression in AC LII/III and V throughout development from P7 to P56. N = 3 mice per age group. Result of 2-way ANOVA: effect of age—*p* = 0.0001 *; effect of region—*p* = 0.0003 *; effect of age X region—0.0082 *. For statistical details, see Table 1.

We also assessed the colocalization of ZnT3 with VGLUT and VGAT in the AC. We observed ZnT3 and VGLUT1 colocalization (Figure 3A–E; Table 1). Furthermore, colocalization in L2/3 was slightly higher than in L5 (Figure 3E; Table 1). Specifically, we observed an increase in VGLUT1 colocalization from P7 (L2/3 = 22.60%; L5 = 9.53%) to P14 (L2/3 = 56.91%; L5 = 54.95%), which increased slightly more by P21 in L2/3 (62.57%) but not in L5 (55.51%, Figure 3E). Similar to the DCN ML, we observed an increase in the ratio of ZnT3 to VGLUT1 in AC L5 (Figure 3F; Table 1), which was also associated with an increase in VGLUT1 expression and colocalization, suggesting that ZnT3 scales with the total number of glutamatergic synapses. In contrast, we observed no change in the ratio of ZnT3 to VGLUT1 over time in L2/3 (Figure 3F), which was associated with increased colocalization, suggesting an increase in the proportion of glutamatergic vesicles containing ZnT3. When we assessed ZnT3 colocalization with VGAT, we observed notable but lower levels of colocalization compared to VGLUT1, which remained stable beyond P14 (L2/3 = 38.95%; L5 = 32.65%; Figure 4A,B and Table 1). Together, between P7 and P14 we observed a developmental increase in ZnT3-HA expression in glutamatergic cortical L2/3 synapses and the emergence of its expression at L5 occur slightly later at P21. Moreover, we observed milder colocalization with GABAergic synapses. Beyond P21, the expression profile of ZnT3-HA remained stable.

**Figure 5:**
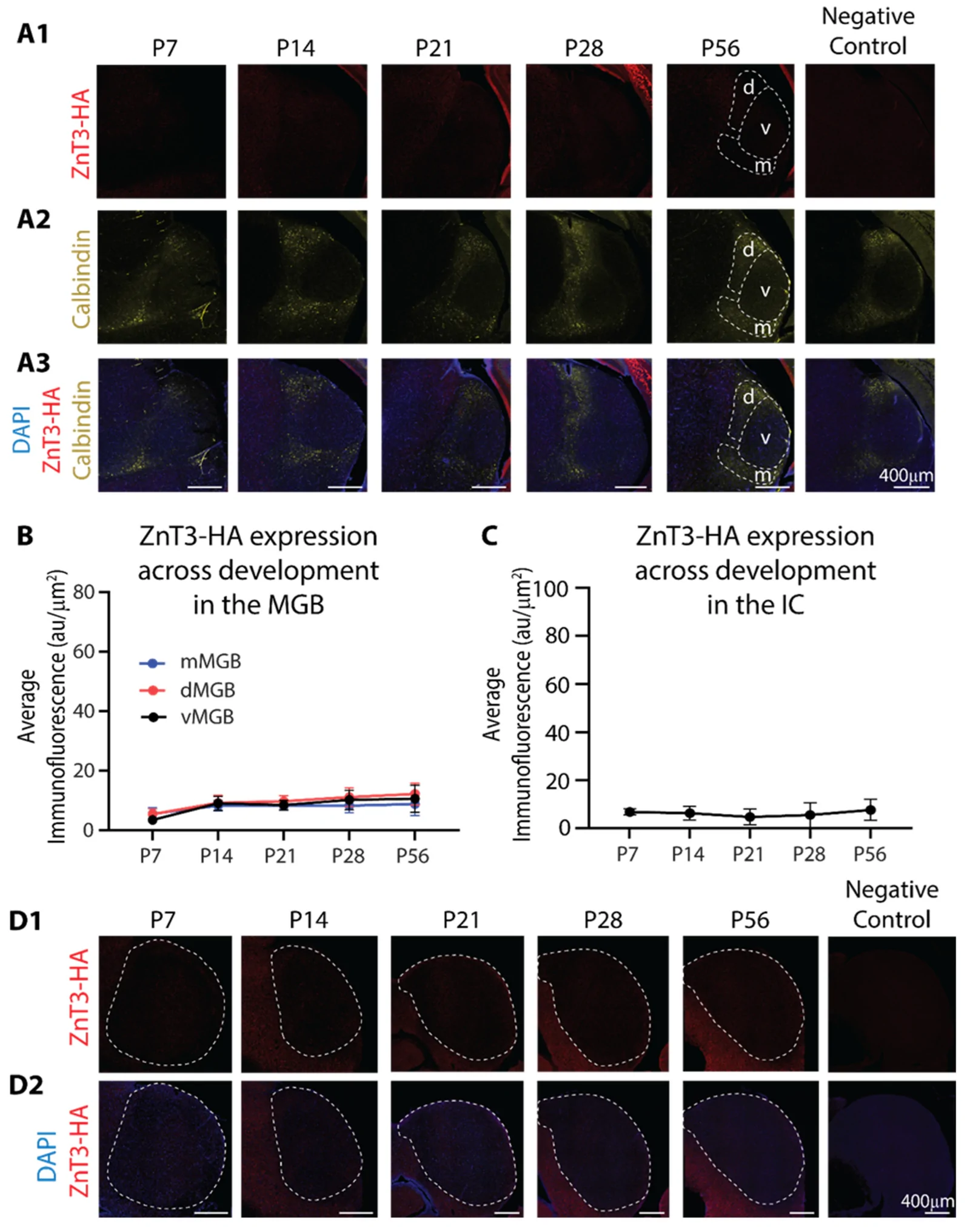
**Expression of ZnT3 in the MGB and IC.** (**A1**) Representative low-magnification (20×) images of ZnT3-HA (red) staining in the MGB throughout development from P7 to P56. Negative control image from P56 mouse on far right. (**A2**) Representative low-magnification (20×) images of Calbindin (yellow) staining in the MGB throughout development from P7 to P56. This stain was used to identify the subregions of the medial geniculate body. Negative control image from P56 mouse on far right. (**A3**) Composite representative low-magnification (20×) images of ZnT3-HA, Calbindin, and DAPI (blue) staining in the MGB throughout development from P7 to P56. Negative control image from P56 mouse on far right. Scale bar = 400 µm. (**B**) In reference to (**A1**). Quantification of average immunofluorescence of ZnT3-HA expression in the subregions of the MGB throughout development from P7 to P56. N = 3 mice per age group. (**C**) In reference to (**D1**). Quantification of average immunofluorescence of ZnT3-HA expression in the IC throughout development from P7 to P56. N = 3 mice per age group. (**D1**) Representative low-magnification (20×) images of ZnT3-HA staining in the IC throughout development from P7 to P56. Negative control image from P56 mouse on far right. (**D2**) Composite representative low-magnification (20×) images of ZnT3-HA and DAPI staining in the inferior colliculus throughout development from P7 to P56. Negative control image from P56 mouse on far right. Scale bar = 400 µm.

Next, we assessed ZnT3 expression in the IC. We did not observe any ZnT3 expression in the IC, consistent with previous studies showing no vesicular zinc staining in the IC (Figure 5C,D) [23]. We next examined the expression of ZnT3 in the auditory thalamus, the medial geniculate body (MGB). Previous studies have reported that a small subset of auditory thalamocortical MGB neurons express ZnT3, which modulates NMDAR currents in L5 pyramidal tract neurons [26]. However, we observed negligible expression of ZnT3 in the MGB (Figure 5A,B; Section 4). Because of the negligible ZnT3 signal in both the MGB and IC (Section 4), we did not perform colocalization studies.

## 4. Discussion

**Figure 6:**
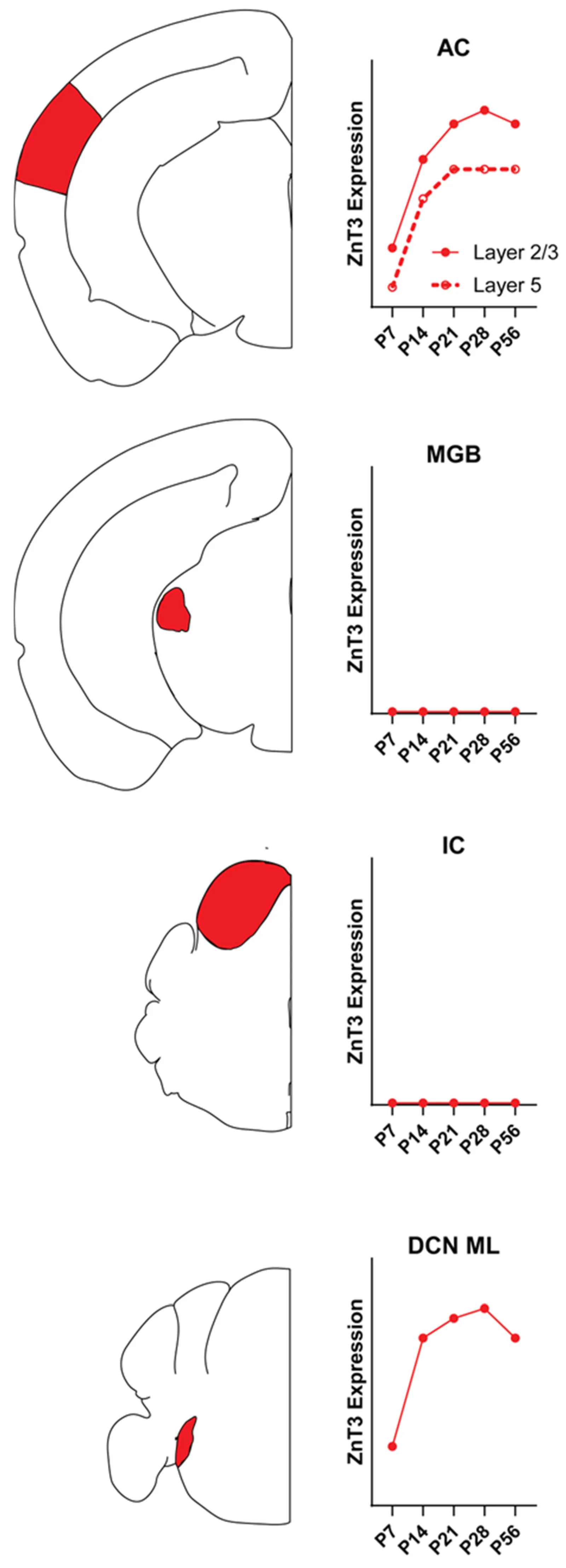
Summary of developmental expression of ZnT3 across the auditory system.

Our results demonstrate a developmental increase in ZnT3 expression levels in the first few postnatal weeks. Namely, ZnT3 expression in both the DCN and AC increase significantly between P7 and P14, reaching a stable overall expression level and distribution by P21 that is maintained through P56 (Figure 1, Figure 2, Figure 3 and Figure 4, summarized in Figure 6). The increase from P7 to P14 corresponds to the onset of hearing in mice, which occurs around P12 [35]. Therefore, the increase in ZnT3 and synaptic zinc could be related to an overall increase in the peripheral input to the auditory system. By P28, when the auditory system is thought to be largely mature, synaptic zinc signaling also appears to be in its mature state.

Previous studies have suggested that ZnT3 protein expression is delayed relative to the emergence of presynaptic zinc in the developing barrel cortex [36]. Therefore, while the ZnT3 expression patterns observed here are consistent with adult ZnT3 expression, we cannot rule out the possibility that the ZnT3 protein expression in P7–P14 mice is not associated with identical changes in synaptic zinc levels. Namely, synaptic zinc accumulation may precede or differ from ZnT3 expression at these early ages. Indeed, while in the adult mouse brain *Znt3* mRNA is tightly correlated with vesicular zinc, prior to P12 vesicular zinc is not well correlated with *Znt3* mRNA expression [37], suggesting that vesicular zinc may be reliant on other zinc transporters. Additionally, in the developing visual thalamus of rats, synaptic zinc can be only transiently detected during synaptic remodeling and is entirely absent beyond P21 [38]. Therefore, it is possible that the snapshot timepoints used in our experiments may have missed brief periods of developmental changes in ZnT3 expression.

Our data demonstrate colocalization of ZnT3 with both VGLUT1 and VGAT in AC. Previous work has shown that ZnT3 is overwhelmingly found in VGLUT1-positive vesicles, and ZnT3 is infrequently found in vesicles with transporters for other neurotransmitters [7]. On the other hand, multiple studies have demonstrated that *Znt3* mRNA is expressed by GABAergic neurons and that synaptic zinc influences GABAergic neurotransmission [13,26,39]. Specifically, around half of all parvalbumin- and somatostatin-positive neurons in AC also express *Znt3* mRNA [26]. Moreover, previous work has demonstrated that synaptic activation of GABAergic somatostatin-positive (SOM) interneurons leads to synaptic zinc-mediated modulation of GABAergic inhibitory postsynaptic potentials in the AC [13], supporting that ZnT3-dependent zinc signaling is critical for fine-tuning GABAergic neurotransmission. Consistent with this, we found that ZnT3 colocalized with ~50% of VGAT immunolocalization in DCN and AC. However, only around 10% of the overall ZnT3 signal is colocalized with VGAT (Figure 2A,C). These results indicate that, while many GABAergic synapses contain ZnT3, this constitutes a small proportion of the synapses that contain ZnT3. Furthermore, a technical limitation of the resolution of our imaging approach is that we cannot rule out that some of the observed ZnT3 co-expression with VGAT could represent VGLUT1/VGAT colocalization. As we did not simultaneously triple label the tissue with antibodies for VGLUT1, VGAT, and ZnT3-HA, we cannot distinguish between ZnT3 co-expression with VGAT versus ZnT3 co-expression with VGLUT1 that also is co-expressed with VGAT. Future studies utilizing higher-resolution or electron microscopy will be required to determine co-expression of ZnT3 with other transporters at the vesicular level.

Here, we observed negligible expression of ZnT3 protein in the MGB (Figure 5). However, our previous findings support that some MGB neurons express the *Slc30a3* (ZnT3) gene, and stimulation of thalamocortical inputs on the superficial cortical layers modulate zinc-mediated inhibition of NMDAR currents of L5B extratelencephalic (ET) neurons in a ZnT3-dependent manner [26]. Taken together, these findings are consistent with the notion that ZnT3 is transported to thalamocortical axon terminals in the AC L5 apical dendrites, with negligible presence in the MGB somata.

To our knowledge, there are no previous reports of synaptic zinc modulation at synapses located within the IC, which is consistent with the lack of ZnT3 protein expression in these locations. Nonetheless, we do not know the absolute detection limits of HA-tag immunofluorescence; thus, while our results are consistent with other methods, such as Timm staining and in situ hybridization and electrophysiology, we cannot rule out the possibility that null results in the MGB and IC are not affected by the HA tag detection limits.

We have not yet performed a highly detailed characterization of either ZnT3-HA protein sub-cellular localization or protein–protein interactions. However, the ZnT3-HA construct was designed to add the HA epitope tag to the carboxy (C) terminus of the endogenous ZnT3 protein. This is important, as previous studies indicated that the addition of an epitope tag to the C-terminus maintained ZnT3 protein localization to vesicles [40,41], supporting that the HA tag does not influence either sub-cellular localization or ZnT3 protein–protein interactions. Moreover, the expression levels of the ZnT3-HA protein are not expected to be different from ZnT3 levels, as the mouse line was designed using Crispr/CAS9 with the epitope tag knocked-in to the endogenous ZnT3 locus. Importantly, we have validated this mouse in our previous work [18]. Namely, to confirm that ZnT3-HA mice accumulate vesicular zinc in the same regions as wildtype (WT) mice, we performed zinc autometallography (also called Timm stain) to stain labile zinc in brain slices from ZnT3-HA mice. We observed that labile zinc accumulation in ZnT3-HA mice is similar to the ZnT3-HA expression patterns in ZnT3-HA mice, in both intensity and distribution pattern; this pattern matches the known labile zinc distribution found in WT mice. Crucially, the same study supports that the addition of the HA epitope tag to ZnT3 did not have any obvious impact on auditory function and hearing thresholds, as assessed by auditory brainstem responses. Taken together, these results support that the ZnT3-HA mice provide a good tool for addressing changes in ZnT3 expression and localization.

Vesicular zinc in the auditory system is modulated by sound experience. Following noise exposure, ZnT3 and vesicular zinc levels are significantly increased and mislocalized in the cochlea [18]; and chelation of zinc mitigates the noise-induced hearing loss [18]. Moreover, presynaptic ZnT3-dependent zinc levels in the DCN are reduced after noise exposure [11,29], although it has not been investigated whether the change in DCN zinc levels are accompanied by a change in ZnT3 protein. In the somatosensory cortex, synaptic zinc is modulated by sensory deprivation [17,42], but the dynamics of ZnT3 expression have remained unknown. However, recent studies using a combination of the ZnT3-HA mouse and genetically encoded zinc sensors showed increased cortical zinc levels associated with increased cortical ZnT3 expression after noise trauma [43], further highlighting the use of the ZnT3-HA mouse in assessing dynamics of ZnT3 expression dynamics during zincergic plasticity. These studies will be important for understanding the roles of synaptic zinc in the pathogenesis and potential treatment of auditory disorders linked to aberrant plasticity after noise trauma, such as tinnitus and hyperacusis [44].

While our study determined the distribution patterns of ZnT3 protein in the auditory system, ZnT3 is highly expressed in other brain areas, including the hippocampus, amygdala, and other cortical areas [20,21,22,23]. Each of these other zincergic brain areas have specific laminar and subregional distributions of vesicular zinc. For example, in the hippocampus, zincergic neurons are found in CA1, CA3, and the dentate gyrus but are absent in CA2 [22]. Moreover, abnormal synaptic zinc signaling has been implicated in animal models of both autism and schizophrenia [45,46,47], and mutations in the *Slc30a3* gene have been identified in humans with schizophrenia [48,49]. Therefore, it will be important to assess how changes in ZnT3 expression correlate with behaviors associated with different brain areas and disorders, as well as sex differences associated with these effects, and whether synaptic zinc signaling is a modifiable factor that could highlight the development of new treatments.

## Figures and Tables

**Table 1:** Statistics table.

Figure	Statistical Test	N (Mice)	Effect of Age	Effect of Region	Interaction of Age × Region	Significant Multiple Comparisons
**Figure 1** **C**	2-way ANOVA	*n* = 3	*p* = 0.2649	*p* < 0.0001	*p* = 0.0658	ML—p7 vs. p21—*p* = 0.0305 ML—p7 vs. p28—*p* = 0.0174 p14—ML vs. DL—*p* = 0.0064 p21—ML vs. DL—*p* = 0.0018 p28—ML vs. DL—*p* = 0.0011 p56—ML vs. DL—*p* = 0.0055
**Figure 1D**	2-way ANOVA	*n* = 3	*p* = 0.9781	*p* < 0.0001	*p* = 0.8180	p14—ML vs. DL—*p* = 0.0481 p21—ML vs. DL—*p* = 0.0192 p28—ML vs. DL—*p* = 0.0099 p56—ML vs. DL—*p* = 0.0496
**Figure 1E**	1-way ANOVA	*n* = 3	*p* < 0.0001	n/a	n/a	p7 vs. p14—*p* < 0.0001 p7 vs. p21—*p* < 0.0001 p7 vs. p28—*p* < 0.0001 p7 vs. p56—*p* < 0.0001
**Figure 2C**	1-way ANOVA	*n* = 3	*p* = 0.7421	n/a	n/a	n/a
**Figure 3C**	2-way ANOVA	*n* = 3	*p* = 0.1430	*p* < 0.0001	*p* = 0.0019	p7—layer IV vs. Layer Va—*p* = 0.0478 p14—Layer II/III vs. Layer IV—*p* = 0.0288 p14—Layer II/III vs. Layer VI—*p* = 0.0361 p21—Layer Va vs. Layer VI—*p* = 0.0145 p28—Layer I vs. Layer IV—*p* = 0.0214 p28—Layer Va vs. Vb—*p* = 0.0306 Layer Va—p7 vs. p21—*p* = 0.0443
**Figure 3D**	2-way ANOVA	*n* = 3	*p* = 0.0454	*p* = 0.0144	*p* = 0.0211	p56—Layer II/III vs. Layer Vb—*p* < 0.0001 Layer I—p7 vs. p28—*p* = 0.0206 Layer II/III—p7 vs. p28—*p* = 0.0408
**Figure 3E**	2-way ANOVA	*n* = 3	*p* < 0.0001	*p* = 0.0001	*p* = 0.0711	p7—Layer II/III vs. Layer V—*p* = 0.0015 Layer II/III—p7 vs. p14—*p* < 0.0001 Layer II/III—p7 vs. p21—*p* < 0.0001 Layer II/III—p7 vs. p28—*p* < 0.0001 Layer II/III—p7 vs. p56—*p* < 0.0001 Layer V—p7 vs. p14—*p* < 0.0001 Layer V—p7 vs. p21—*p* < 0.0001 Layer V—p7 vs. p28—*p* < 0.0001 Layer V—p7 vs. p56—*p* < 0.0001
**Figure 4B**	2-way ANOVA	*n* = 3	*p* = 0.0001	*p* = 0.0003	*p* = 0.0082	p7—Layer II/III vs. Layer V—*p* = 0.0002 Layer II/III—p7 vs. p28—*p* = 0.0164 Layer II/III—p7 vs. p56—*p* = 0.0268 Layer V—p7 vs. p14—*p* < 0.0001 Layer V—p7 vs. p21—*p* < 0.0001 Layer V—p7 vs. p28—*p* < 0.0001 Layer V—p7 vs. p56—*p* < 0.0001

n/a indicates that this factor was not included in the statistical test.

## Data Availability

The original contributions presented in this study are included in the article/Supplementary Material. Further inquiries can be directed to the corresponding authors.

## Supplementary Materials

The following supporting information can be downloaded at https://www.mdpi.com/article/10.3390/cells15171554/s1, Figure S1: HA tag insertion into *Slc30a3* gene. (A)—Wildtype *Slc30a3* gene including 8 exons (gray boxes) and the C-terminal nucleic acid sequence (blue expansion box). (B)—*Slc30a3*-HA (ZnT3-HA mouse) gene including 8 exons and the nucleic acid sequence for the knock in HA tag. Blue nucleic acids correspond to the GSG linker sequence, and the red nucleic acids correspond to the HA tag sequence.
