# Supplementary material for: Developmental Changes in the Protein Expression Levels and Localization of the Vesicular Zinc Transporter (ZnT3) in the Mouse Central Auditory System
Source: Cells. 2026 Aug 27;15(17):1554. doi: 10.3390/cells15171554 (PMC13565375; doi:10.3390/cells15171554)
Supplement: Supplementary file 1 [file cells-15-01554-s001.zip › cells-4429574-supplementary.pdf]

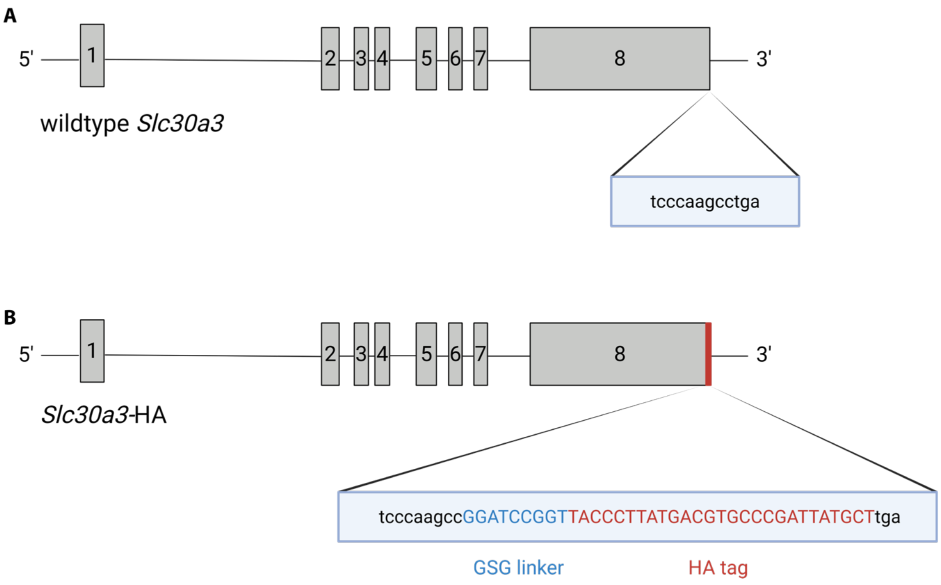

Supplemental Figure 1. HA tag insertion into *Slc30a3* gene. A – Wildtype *Slc30a3* gene including 8 exons (gray boxes) and the C-terminal nucleic acid sequence (blue expansion box). **B** – *Slc30a3*-HA (ZnT3-HA mouse) gene including 8 exons and the nucleic acid sequence for the knock in HA tag. Blue nucleic acids correspond to the GSG linker sequence, and the red nucleic acids correspond to the HA tag sequence.
